# The MFS efflux pump EmrKY contributes to the survival of *Shigella* within macrophages

**DOI:** 10.1038/s41598-019-39749-3

**Published:** 2019-02-27

**Authors:** Martina Pasqua, Milena Grossi, Sara Scinicariello, Laurent Aussel, Frédéric Barras, Bianca Colonna, Gianni Prosseda

**Affiliations:** 1 Istituto Pasteur Italia, Dipartimento di Biologia e Biotecnologie “C. Darwin”, Sapienza Università di Roma, Rome, Italy; 2 Aix-Marseille Univ, CNRS, Laboratoire de Chimie Bactérienne, Institut de Microbiologie de la Méditerranée, Marseille, France; 3 Institut Pasteur, Department of Microbiology, Paris, France

**Keywords:** Bacteriology, Pathogens

## Abstract

Efflux pumps are membrane protein complexes conserved in all living organisms. Beyond being involved in antibiotic extrusion in several bacteria, efflux pumps are emerging as relevant players in pathogen-host interactions. We have investigated on the possible role of the efflux pump network in *Shigella flexneri*, the etiological agent of bacillary dysentery. We have found that *S*. *flexneri* has retained 14 of the 20 pumps characterized in *Escherichia coli* and that their expression is differentially modulated during the intracellular life of *Shigella*. In particular, the *emrKY* operon, encoding an efflux pump of the Major Facilitator Superfamily, is specifically and highly induced in *Shigella*-infected U937 macrophage-like cells and is activated in response to a combination of high K^+^ and acidic pH, which are sensed by the EvgS/EvgA two-component system. Notably, we show that following *S*. *flexneri* infection, macrophage cytosol undergoes a mild reduction of intracellular pH, permitting EvgA to trigger the *emrKY* activation. Finally, we present data suggesting that EmrKY is required for the survival of *Shigella* in the harsh macrophage environment, highlighting for the first time the key role of an efflux pump during the *Shigella* invasive process.

## Introduction

Efflux pumps (EPs) are present in all living organisms and represent an important and consistent group of transporter proteins, which contribute to the resistance to compounds used for treating different diseases^1,2^. In Gram negative bacteria, EPs either form tripartite complexes able to traverse both membranes, including the inner membrane, a membrane fusion protein and an outer membrane protein, or are present as single-component efflux transporter in the inner membrane^2^. According to their sequence similarity, composition, transport function, energy source and substrates, EPs are grouped into five families: the ATP binding cassette (ABC) superfamily, the major facilitator superfamily (MFS), the multidrug and toxic compound extrusion (MATE) family, the small multidrug resistance (SMR) family and the resistance nodulation division (RND) family^1^. The importance of EPs has been associated with their ability to extrude a wide range of antibiotics resulting in the emergence of multidrug resistance in many bacteria, including pathogens^1,2^.

In the last years, several studies have identified numerous functions for EPs that go beyond antibiotic extrusion: these functions range from bacterial interactions with plant or animal hosts, to detoxification of metabolic intermediates and maintenance of cellular homeostasis^3,4^. An interesting aspect concerns the involvement of EPs in the virulence of several bacterial pathogens^3,5^. In enteric pathogens such as *Salmonella Typhimurium*, *Listeria monocytogenes*, and *Vibrio cholera* it has been shown that EPs are relevant virulence factors contributing to persistence and replication in a bile rich environment^6–8^. Moreover, in *Salmonella* EPs are critical for the invasion and survival within macrophages and intestinal epithelial cells and contribute to the different steps of the pathogenicity process^9–11^, while in *Vibrio cholera*, besides favouring the colonization of the intestine, EPs are also required for the full expression of the major virulence determinants^12^. EPs contribute also to the pathogenicity of *Pseudomonas aeruginosa* and *Stenotrophomonas maltophilia*, where they play a significant role in biofilm formation^13–15^, and of *Campylobacter jejuni* and *Neisseria gonorrhea*, where they are involved in the colonization of the host epithelia^16,17^.

*Shigella* is an intracellular pathogen responsible of a life-threatening enteric syndrome in humans^18^. Invasion of the colonic epithelium by *Shigella* is the result of a complex multistep process. After ingestion, *Shigella* gains access to the intestinal mucosa by promoting its uptake into M cells in Peyer’s patches. The bacteria are then released into an intraepithelial pocket and invade resident macrophages, where they multiply and induce rapid cell death. Once released from dying macrophages, invasive bacteria can finally infect the neighboring enterocytes, where they actively replicate and, without any extracellular steps, disseminate from cell-to-cell, causing severe damage and inflammatory destruction of the colonic mucosa^19^.

*Shigella* shares strong homology with its commensal ancestor, *Escherichia coli*, and has derived repeatedly from different branches of the *E*. *coli* tree by convergent evolution involving both gain and loss of genes^20–22^. In particular, the acquisition by horizontal gene transfer of a large plasmid (pINV) carrying genes for a Type III secretion system and its effectors has been the crucial event towards pathogenic lifestyle^19^. This process has been paralleled by the loss of several chromosomal genes unnecessary or deleterious for the invasive process^23–26^.

Very few data are available on the role that EPs might have in the lifestyle of *Shigella* and these are limited to the MdtJI and AcrAB EPs^27,28^. Concerning MdtJI, it has been shown that its expression is higher in *Shigella* compared to *E*. *coli* and is positively controlled by spermidine and by the VirF protein, the major regulator of the *Shigella* invasive genes^29^. On the basis of its ability to secrete putrescine, it has been proposed that, maintaining the spermidine at an optimal level, MdtJI might contribute to the *Shigella* survival within the host cells^27,30^. In the case of AcrAB it has been shown that also in *Shigella* it contributes to bile salts resistance^28^.

Taking into account the emerging role of EPs in bacterial virulence and the importance of *Shigella* as human pathogen we asked whether EPs might be involved in *the Shigella* mediated invasive process. To this end, we searched for the EPs conserved in the *Shigella flexneri* genome and then investigated whether the expression of their encoding genes was influenced by the host cell environment. Remarkably, we found the *emrKY* genes, enconding the MSF efflux pump EmrKY, to be strongly activated in macrophages. The up-regulation of the *emrKY* expression in the macrophage environment led us to investigate further the regulatory mechanism underlying its specific activation and determine its potential role during *Shigella* intracellular life.

## Results

### *In silico* identification of the *Shigella flexneri* EP encoding genes

*E*. *coli* synthesizes 20 functional EPs^31,32^. By using the NCBI genome BLAST, homologs for each of the 20 *E*. *coli* EP encoding operons were searched in the genome of *S*. *flexneri* M90T, a strain widely used in laboratory to analyze *Shigella*-host interactions^33^. As shown in Table 1, only 14 out of the 20 genetic systems described in *E*. *coli* are conserved in *S*. *flexneri*. A more detailed inspection of the rearrangement that have caused the silencing of six EP encoding loci in M90T, revealed that three of them (*cusCFBA*, *mdtABCD*, *mdtEF)* were disrupted by the insertion of IS elements within the coding or regulatory regions, while the remaining three were completely lost (*acrEF*, *yceE* and *yjiO*). The 14 EP encoding genes identified in M90T strain are conserved in all the other *S*. *flexneri* genomes deposited in NCBI database, suggesting a potential involvement in *Shigella* virulence.

**Table 1:** Analysis of the efflux pump encoding genes present in the *S*. *flexneri* M90T genome.

Family	*E*. *coli*	*S*. *flexneri*
ABC	*macAB*	+
MFS	*bcr*	+
	*emrAB*	+
	*emrD*	+
	*emrKY*	+
	*Fsr*	+
	*mdfA*	+
	*mdtG* (*yceE*)*	−
	mdtH (yceL)*	+
	mdtL(yidY)*	+
	mdtM (yjiO)*	−
RND	*acrAB*	+
	*acrD*	+
	*acrEF*	−
	*cusB*	NF
	*mdtABC*	NF
	*mdtEF*	NF
SMR	*emrE*	+
	*mdtJI*	+
MATE	mdtK (ydhE)*	+

Functional efflux pumps conserved or silenced in the *S*. *flexneri* genome. The 20 genetic systems encoding functional efflux pumps in *E*. *coli* have been selected according to Kobayashi *et al*.^32^. The comparative analysis between the genome of *E*. *coli* K-12 and *S*. *flexneri* M90T strain was carried out using NCBI BLAST (http://blast.ncbi.nlm.nih.gov/Blast.cgi). The nucleotide sequences of each efflux pump encoding gene were obtained from EcoCyc (https://ecocyc.org).

^+^Efflux pump encoding genes present in the *S*. *flexneri* genome (sequence homology ≥92%).

^−^Not present in the *S*. *flexneri* genome.

NF: pseudogene, i.e. not Functional due to gene disruption.

^*^The denomination in brackets corresponds to the former one adopted in *E*. *coli* and is still in use in *S*. *flexneri*.

### Expression profile of the *Shigella* EP encoding genes during infection of host cells

**Figure 1:**
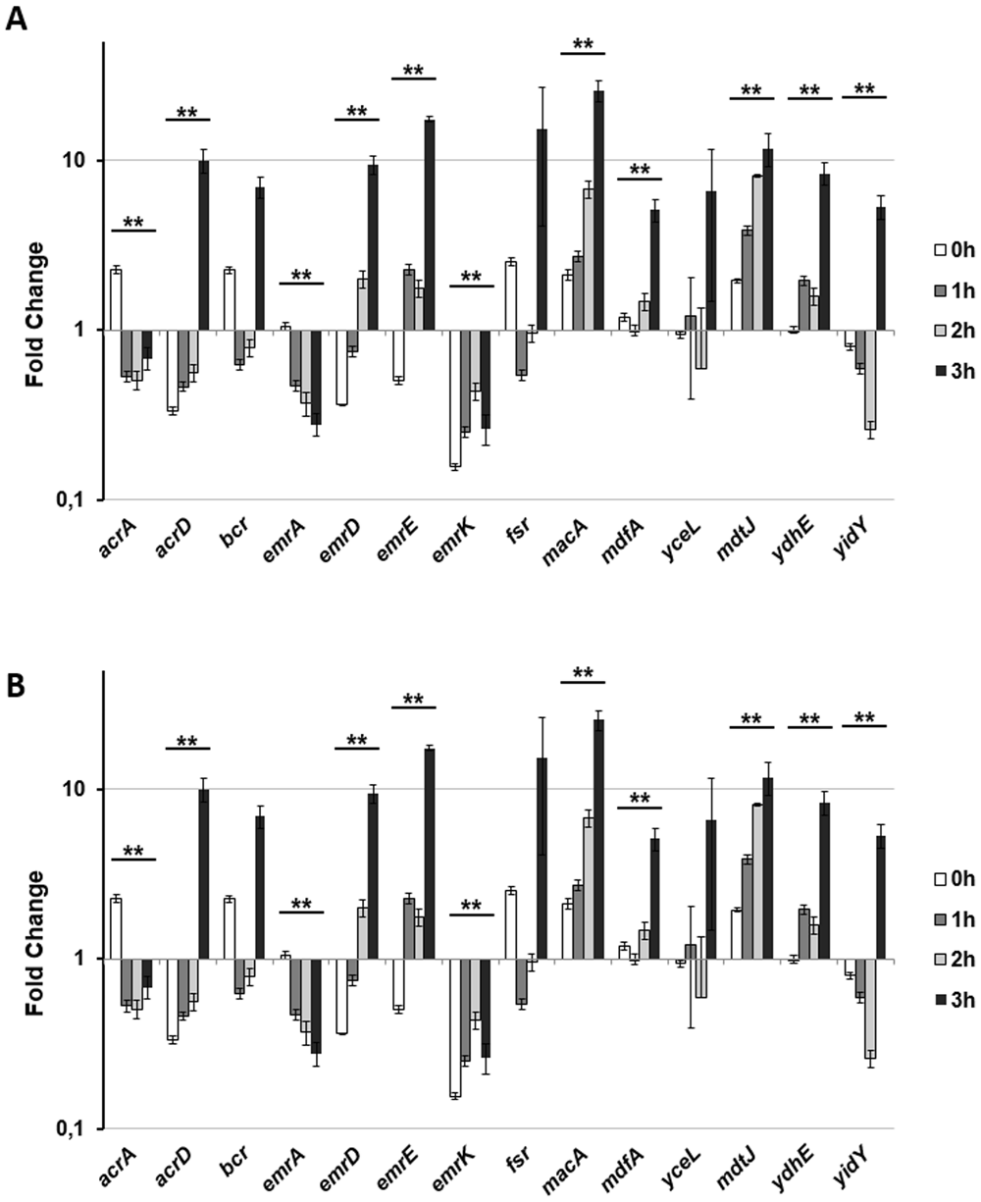
Relative efflux pumps encoding gene transcription during *S*. *flexneri* infection of (**A**) U937 and (**B**) Caco-2. Quantitative analysis of 14 efflux pump transcripts was performed by means of Q-Real Time PCR assay. Total RNA was extracted from intracellular *S*. *flexneri* M90T bacteria at various time points p.i., from 0 h (corresponding to bacterial adhesion to the target cells, see MM) up to 3 h. Each infection was repeated three times and at least three wells were run for each sample. The x axis indicates the expression fold-change (RQ value) for each gene on a logarithmic scale. The results are shown relative to the expression of each gene in bacteria grown in RPMI (A) or DMEM (B) set to 1.00. Statistical significance was determined by a one-tailed ANOVA, and p values are as follows: *p < 0.05, **p < 0.01. Error bars represent SD.

Transcription profile of the *S*. *flexneri* EP genes was assessed during infection of human cells. *Shigella* pathogenicity process is characterized by the ability of bacteria to overcome the macrophage attack and subsequently to invade the epithelial cells. Thus, we first analyzed the expression profile of the EP encoding genes in *Shigella*-infecting human U937 monoblasts differentiated into macrophage-like cells. As shown in Fig. 1A, expression of some EP genes increased when *Shigella* invades macrophages as compared to growth in RPMI medium. In particular, transcription of the *emrK* gene was induced more than 30-fold with its expression steadily increasing during infection. Similarly, transcript levels of *emrA*, *emrD* and *emrE* genes increased significantly during *Shigella* infection (up to 2, 8 and 10- fold, respectively) following similar kinetics. Expression of *mdtJ* gene was also induced, while in this case, maximum level was reached almost immediately upon entry into the macrophage (up to 7-fold). Conversely, expression of *acrA* gene was found to be down-regulated in the macrophage environment, whereas expression of remaining EP encoding genes was not significantly changed going from growth into RPMI medium to macrophage.

Expression level of the *S*. *flexneri* EP genes was also analysed during infection of Caco-2 epithelial cell line. Surprisingly, expression of both *emrK* and *emrA* genes was down-regulated, in contrast to what had been observed in macrophages (Fig. 1B). Expression of the *macA* gene was strongly induced in Caco-2 cells, whereas it had been found unaltered inside macrophages. Last, a series of genes showed the same pattern of expression in both cell lines with the *mdtJ* and *ydhE* genes being up-regulated and *acrA* gene being down-regulated. Also, *emrD* and *emrE* are induced in Caco-2 cells although at later time during infection.

In conclusion, transcript analyses indicated that expression of a subset of the 14 conserved EP-encoding genes varies depending upon the host cell type *Shigella* is growing in. Entry and subsequent killing of macrophages represents the rate limiting step of the *Shigella* invasive process^34^. Given the cell specific expression of the *emrK* gene, belonging to the *emrKY* operon, we focused our subsequent efforts on the regulation and potential role of this EP within macrophages.

### Expression of *emrKY* in response to environmental stimuli

**Figure 2:**
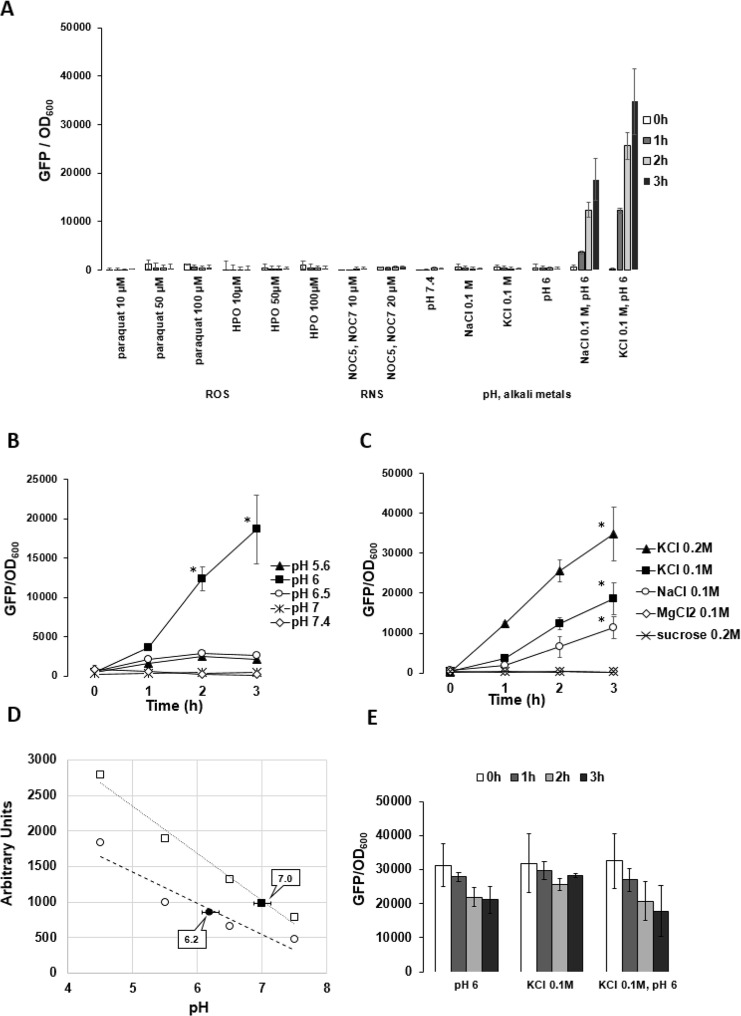
e*mrKY* expression in *S*. *flexneri* is modulated by specific stimuli both outside and inside U937. (**A**) Expression of *emrKY* in response to the indicated stimuli in strain M90T p*ZemrK-gfp* grown in LB or M9 complete medium. (**B**) Expression of *emrKY* in response to different pH values (from pH 5.6 to 7.4) in M9 complete medium supplemented with 0.1 M KCl. (**C**) Expression of *emrKY* in response to increasing concentration of KCl, NaCl, MgCl_2_ or sucrose added to M9 complete medium at pH 6. The addition of 0.05 M MgCl_2_ or 0.1 M sucrose (not shown) has the same effect as 0.1 M MgCl_2_ or 0.2 M sucrose, respectively. In A,B and C the expression of *emrKY* was measured as a function of GFP production in M90T p*ZermK*-*gfp* strain. (**D**) Intracellular pH values (filled symbols) of mock (square) and M90T infected U937 cells (circle) at 3 hours p.i. pH was measured by loading cells with green pHrodo dye and quantified on the relative standard curves obtained with four different calibrated pH buffers. Fluorescence intensity was read on the Victor multilabel counter (Perkin Elmers). (**E**) Expression of *mdtJI* in response to the indicated stimuli measured as a function of GFP production in M90T p*ZmdtJ-gfp* strain grown in M9 complete medium. GFP production was evaluated by reading fluorescence intensity on Infinite 200 (TECAN) every 15 minutes throughout 3 hours. GFP values were normalized to the bacterial cell concentration and the results shown are the mean of three independent experiments. Three independent experiments were carried out and statistical significance was determined by a one-tailed ANOVA, and p values are as follows: *p < 0.05. Error bars represent SD.

We aimed at identifying the stimuli causing *emrKY* induction in macrophages. *Shigella* infecting a macrophage faces oxidative stress, nitrosative stress and acidic environment. Therefore, we used paraquat and hydrogen peroxide (HPO) to induce the production of reactive oxygen species (ROS), and the NO-amine complexes NOC-5 and NOC-7 to induce the formation of reactive nitrogen species (RNS), while acidic conditions were achieved by adding HCl to M9 complete medium. We also evaluated whether an unbalanced composition in alkali metals (NaCl or KCl) acted as a stimulus. To efficiently monitor *emrKY* expression, we cloned the *gfp* gene under the control of the *emrKY* promoter and transformed the resulting plasmid p*ZemrK-gfp* into M90T. As shown in Fig. 2A when paraquat, HPO, NOC-5 or NOC 7 was added to a culture of M90T p*ZemrK-gfp* at OD_600_ 0.2, expression of GFP remained close to the basal level. Similarly, neither medium acidification nor the presence of alkali metals modified the expression level of the reporter gene (Fig. 2A). In contrast, a very high level of GFP was observed when the culture was grown at pH 6.0 in M9 complete medium supplemented with KCl or NaCl (0.1 M). Considering that M9 medium already contains 0.093 M NaCl and only 0.022 M KCl, a lower final concentration of KCl is sufficient to obtain a more effective induction (Fig. 2A).

Interestingly, when the pH value of the M9 medium was adjusted at different pH values from pH 5.6 to pH 7.4, stimulatory effect on the *emrKY* promoter was observed only at pH 6 (Fig. 2B). The addition of KCl (0.2 M) to the M9 medium yielded a two-fold enhancement in the *emrKY* expression (Fig. 2C). The role played by high concentrations of K^+^ and Na^+^ ions is evidenced by observing that no induction occurs when another chloride salt, MgCl_2_ (0.05 M and 0.1 M) is used, thus excluding a potential involvement of Cl^-^ ions in agreement with previous observations^35^. Last, the fact that addition of MgCl_2_ (0.1 M) or sucrose (0.1 M or 0.2 M) has no effect on *emrKY* induction (Fig. 2C), indicated that the *emrKY* up-regulation, observed with KCl or NaCl, was not due to an overall increase in osmolality.

To test whether pH 6 and high concentration of K^+^ ions recapitulated part of the physico-chemical environment met by *Shigella* in macrophages, we measured the macrophage intracellular pH (pHi) during bacterial infection. The data shown in Fig. 2D demonstrate that, upon *Shigella* infection U937 pHi value dropped to 6.2, whereas mock infected cells maintained a neutral pHi value. The values reported refer to 3 hours post infection (p.i.), when the infection efficiency was at its peak, ranging from 60 to 70% in the various infection experiments.

Interestingly, *mdtJ* gene, whose expression is induced in both macrophage and epithelial cells, was not modified in M9 complete medium supplemented with 0.1 M KCl and set at pH 6 (Fig. 2E), leading some credence to the notion that EmrKY carries on specific tasks in the macrophage compartment.

Finally, we asked whether in the macrophage environment the high expression of *emrKY* was linked to a reduced expression of *acrAB*, encoding the major *E*. *coli* EP^31^. Indeed, it has been suggested that the loss of a given EP might be compensated by an increased synthesis of another EP that could fulfill, at least in part, the same function^36,37^. We generated M90T derivatives lacking the EmrKY (M90T Δ*ermKY*) or AcrAB (M90T Δ*acrAB*) systems or overproducing the AcrAB pump (M90T *pACacrAB*). U937 cells were infected with M90T or its derivatives, and the *acrA* or *emrK* gene transcripts analyzed at different time points p.i. As reported in Fig. S1, induction of *emrK* gene was influenced neither by the absence nor by the overexpression of *acrAB* genes. Conversely, presence or absence of *ermKY* has no effect on the *acrA* transcription level.

### *Shigella emrKY* is regulated by EvgA both in laboratory conditions and within host cells

**Figure 3:**
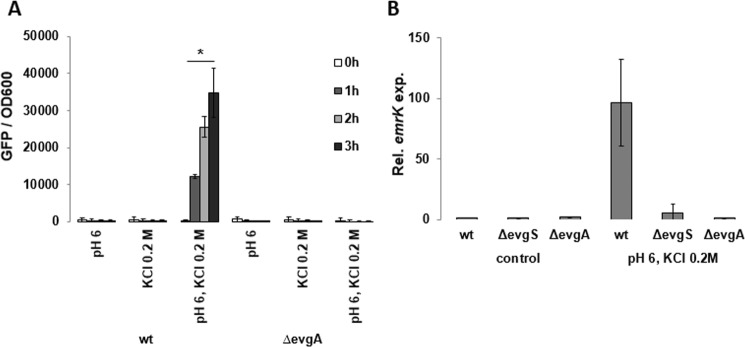
Regulation of *emrKY* expression in *S*. *flexneri*. (**A**) Expression of *emrKY* was monitored as a function of GFP production in *S*. *flexneri* M90T and its derivative lacking the EvgA regulator (M90T*ΔevgA)*, both strains carrying the p*ZemrK-gfp* plasmid. Strains were grown in M9 complete medium modified as indicated. GFP production was monitored by reading the fluorescence intensity on Infinite 200 (TECAN) every 15 mins throughout 3 h. (**B**) Quantitative analysis of the *emrK* transcript was performed by qRT-PCR using RNA extracted from *S*. *flexneri* M90T and its derivatives lacking the EvgA (M90T Δ*evgA*) or EvgS (M90T Δ*evgS*) proteins. All *S*. *flexneri* strains were grown at 37 °C in M9 complete medium at pH 7.4 (control) or in M9 at pH 6 supplemented with 0.2 M KCl (induction conditions). The experiment was repeated three times and at least three wells were run for each sample. The results are shown relative to the *emrK* transcript levels in M90T grown in control conditions set to 1.00. Statistical significance was determined by a one-tailed ANOVA, and p values are as follows: *p < 0.05. Error bars represent SD.

In *E*. *coli*, the *emrKY* operon is under the control of the EvgS/EvgA two-component system^38,39^ in response to mild acidic conditions and high concentrations of alkali metals^35,40^. Therefore, we tested whether this was also the case in *Shigella* both in laboratory conditions and in host cell during infection. We introduced a deletion covering the *evgA* gene in the M90T and monitored the *emrK-gfp* expression. Lack of *evgA* prevented *emrK-gfp* fusion expression under inducing conditions (pH 6.0, 0.2 M KCl) (Fig. 3A). Consistently, no *emrK* transcrips was evidenced in strains lacking either EvgA (M90T Δ*evgA*) or EvgS (M90T Δ*evgS*) grown in inducing conditions (Fig. 3B).

**Figure 4:**
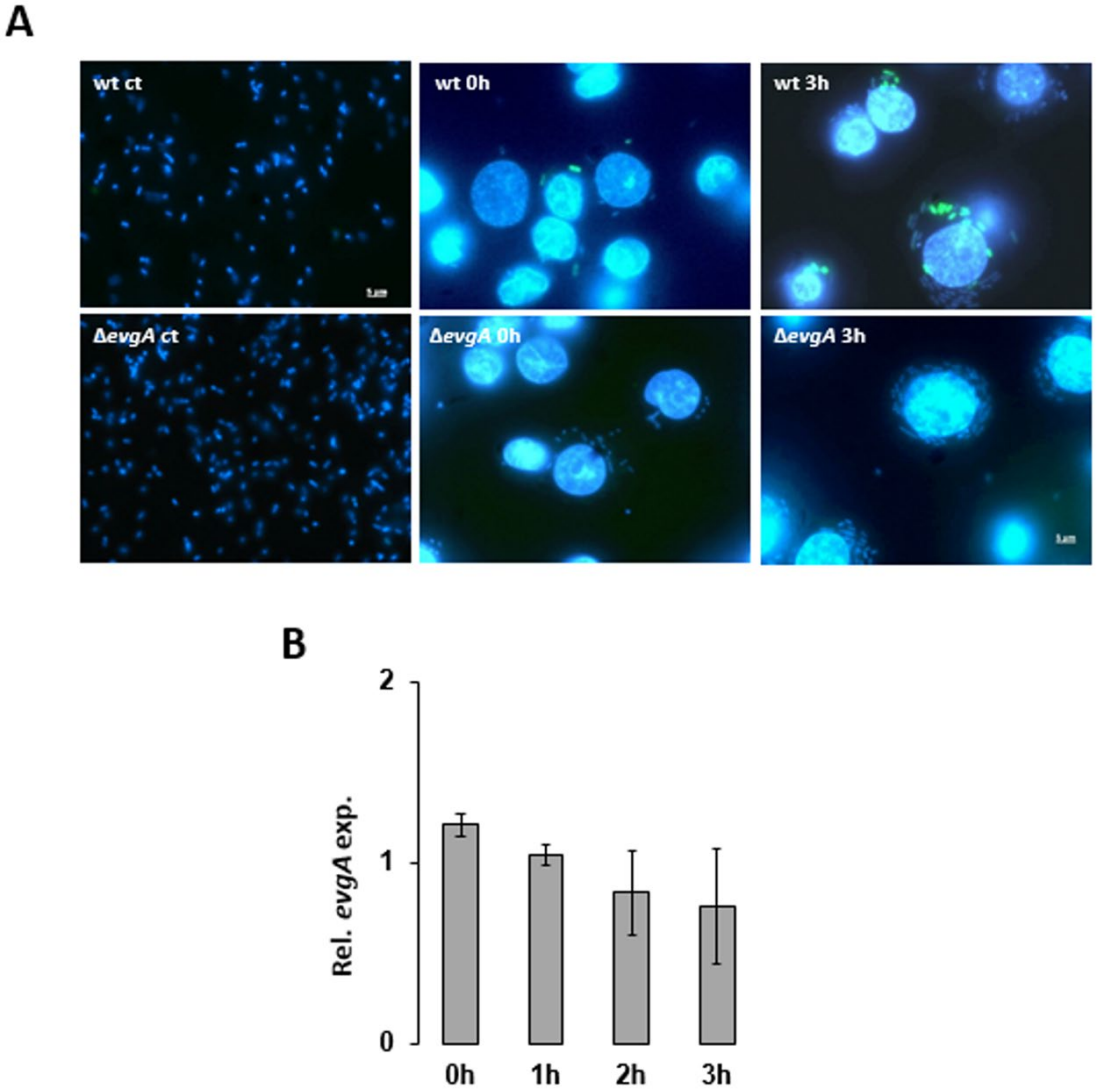
*EmrKY* induction within macrophages is under the control of EvgA. (**A**) Fluorescence microscopy images of *S*. *flexneri* M90T and M90T Δ*evgA* strains (WT ct and Δ*evgA* ct), harboring the *emrK*-*gfp* fusion, grown in laboratory conditions (first column) and during the infection of U937 cells at 0 h (second column) and 3 h p.i. (third column). Bacteria and macrophage DNA was stained with DAPI. Individual pictures of the same field, taken with a DC camera, were merged using a Leica Microsystem Imaging Equipment. (**B**) Quantitative analysis of the *evgA* transcript from M90T in U937 cells at the indicated time points. The experiment was repeated three times and at least three wells were run for each sample. The results shown are relative to the *evgA* transcript levels in M90T grown in M9 (pH 7.4) set to 1.00. Statistical significance was analyzed by a one-tailed ANOVA. Error bars represent SD.

To test whether induction of *emrKY* is mediated by EvgA in the macrophage environment, we infected U937 cells with M90T or its Δ*evgA* derivative, both carrying the pZ*emrK-gfp* plasmid. As shown in Fig. 4A expression of *emrK-gfp* fusion occurred very early in the M90T strain during the infection process and was maintained throughout the infection period. In contrast, the M90T Δ*evgA* mutant was unable to activate the *emrKY* promoter as indicated by the absence of intracellular green bacteria at all the time points analyzed. Importantly, *evgA* expression was no significantly modified in macrophage environment (Fig. 4B).

Altogether, these data demonstrate that the *Shigella emrKY* operon is under the positive control of the EvgS/EvgA two-component system in response to mild acidic and high K^+^ concentration conditions met in macrophage environment.

### EmrKY contributes to *Shigella* fitness in macrophages

**Figure 5:**
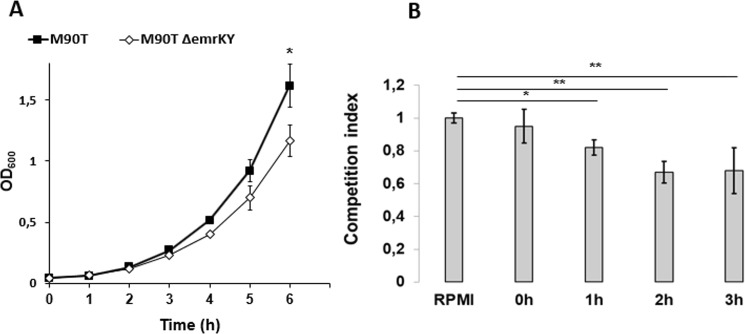
*EmrKY* confers a growth advantage to *Shigella* and improves its survival inside macrophages. (**A**) Growth curves (OD_600_) of M90T and its derivative lacking the EmrKY pump (M90T*ΔemrKY*), in M9 complete medium pH 6.0 supplemented with 0.2 M KCl. (**B**) Competitive indices (C.Is), at the indicated time points p.i., of M90T *ΔemrKY* relative to M90T wt strain as normalized by their ratio in the inoculum (RPMI). M90T and M90T*ΔemrKY* were used to coinfect U937 cells at MOI 100. The data presented are the means of three independent experiments. Statistical significance was determined by t test, and p values are as follows: *p < 0.05. Error bars represent SD.

Since induction of *emrKY* genes is part of the bacterial response to peculiar conditions (moderate acid conditions and unbalanced alkali metals) it is reasonable that EmrKY can confer a growth advantage in those same conditions and can be required to better face the hostile environment. To verify this hypothesis, we compared the growth properties of *S*. *flexneri* M90T and its Δ*emrKY* derivative at pH 6.0 in M9 complete medium supplemented with 0.2 M KCl. Figure 5A shows that, actually, lack of EmrKY negatively affected the bacterial growth, suggesting that induction of this EP contributes to resist to this adverse environment (Fig. 5A). Following the same line, we asked whether EmrKY pump enhanced survival of *Shigella* in macrophages. To this end, we set up a competitive assay where U937 cells were co-infected with the M90T and its Δ*emrKY* derivative. Intracellular bacteria were recovered, counted and the competitive index was determined at various time points after infection (see Methods). The data reported in Fig. 5B clearly demonstrate that the M90T strain exhibited an intracellular growth advantage compared to its Δ*emrKY* derivative. Indeed, the competitive index related to the Δ*emrKY* strain decreased progressively reaching 0.67 at two hours p.i., a value which was not significantly affected by an additional incubation of 1 hour. This indicates that increasing expression of *emrKY* (Fig. 1A) triggered by U937 cytoplasm acidification (Fig. 2D) is functional for *S*. *flexneri* survival and multiplication within macrophage environment. In conclusion, these data support the notion that the massive presence of EmrKY during *Shigella* infection of macrophages could represent one of the multiple bacterial strategies to overcome host defenses.

## Discussion

In the present study, we report that many of the *S*. *flexneri* EP genes have their expression differentially modulated during the intracellular life. Notably, our findings highlight how the expression of some EPs is regulated by specific cell context. A most telling case is that of the EmrKY pump, the genes of which are highly and specifically activated in *Shigella*-infecting U937 macrophage-like cells. We demonstrated that the *Shigella emrK* gene is induced in response to a combination of high K^+^ and mild acidic pH. Both of these physico-chemical features were found to be sensed and transduced by the EvgS/EvgA two-component system, both in laboratory conditions and in infected macrophages. Last, evidence was obtained showing that EmrKY is functional to *Shigella* to better overcome the macrophage obstacles, pointing out the pivotal role of an EP in *Shigella* invasive process.

The pathogenesis of *Shigella* is extremely complex and is based on its capability to cross the intestinal barrier, to kill resident macrophages and invade enterocytes, where intracellular replication and dissemination occurs^19^. *Shigella* shares a high genome homology with the commensal *E*. *coli* and on the basis of phylogenetic analysis *Shigella* has been included in *E*. *coli* species^20,21^. However, compared to *E*. *coli*, *Shigella* genome is extremely dynamic and subjected to an extensive gene decay process, which has targeted widely diverse housekeeping functions such as catabolic pathways or mobility^24^. Here we report that gene decay has targeted EP genes as only 14 out of 20 were conserved. In turn, this advocate for these 14 to have a role in *Shigella* physiology and adaptation, possibly in relation with its intracellular lifestyle in the human host.

Besides antibiotic resistance, bacterial EPs likely take part in several relevant processes of the microbial physiology, including bacteria-host interactions^1,3,5^. Involvement of EPs has been described in several pathogenic bacteria where they contribute to host colonization, intracellular survival, resistance to stress and biofilm formation^3–5^. In *Shigella*, the role of EPs in the intracellular life have been very poorly investigated. In the case of MdtJI, an EP involved in polyamine trafficking, it has been hypothesized that its activity in secreting polyamines might be associated with an increased survival within the host cells^27^. In the present study, transcription profile analysis of EP genes during *Shigella* infection showed that they are differentially expressed according to the cell environment. Of particular interest was that some of them were found up-regulated upon U937 macrophage infection. Escape from macrophage being considered as the rate limiting step in *Shigella* infection, the reprogramming of EP synthesis might be crucial within the overall invasive process.

EmrKY stood out as the most up-regulated EPs in *Shigella*-infecting macrophages. EmrKY is an efflux pump composed by an inner membrane subunit and a periplasmic adaptor protein belonging to the MF superfamily^1^. The structure of the EmrKY pump has not yet been solved. Comparative studies suggest that EmrKY shares homology with another MSF efflux pump, EmrAB^41,42^. In contrast to EmrAB, EmrKY does not contribute to antibiotic resistance but when overexpressed confers the cell resistance to bile salts^31^. Finally, loss of EmrKY reduces the ability of *E*. *coli* cells to form biofilm^43^ and increases their sensibility to DNA damaging compounds^44^.

In *E*. *coli*, the *emrKY* operon is regulated in response to K^+^ and mild acidic pH via the EvgS/EvgA two-component system^35^. Here we found the same applied to the *Shigella emrKY* operon. Importantly, this very regulatory circuit also takes place in the host as mutants defective in *evgA* were unable to activate transcription of *emrK* inside macrophages.

*Shigella emrKY* operon was found to be regulated in an opposite manner in macrophage and epithelial cells, as it was induced in the first case and repressed in the second. Taking into account the stimuli able to upregulate the *emrKY* operon, it can be envisaged that infected macrophage and epithelial cell environment differs markedly in some features. For what it concerns K^+^, it is well known that one important function of animal cells is to maintain an ion gradient across the plasma membrane and, for example, the Na^+^- K^+^ pump ensures the maintenance of high K^+^ and low Na^+^ intracellular concentration^45^. Thus, the relative high cytosolic levels of K^+^ could mirror the high K^+^ concentration required to induce the *emrKY* operon in laboratory conditions. However, this doesn’t explain the opposite expression profile of *emrKY* within macrophage and epithelial cells, as such an ion gradient is present in both cell types. Another explanation might stem from considering the pH factor. *Shigella* could face completely different cytosolic pH conditions in macrophage and epithelial cells. Indeed, Lucchini and collaborators^46^, analyzing genomic expression of *S*. *flexneri* during the infection of macrophage and epithelial cells, found that genes involved in acid resistance were strongly induced in U937 cells but not in HeLa cells, suggesting that, within macrophages, *Shigella* is exposed to an acidic cytosol. Accordingly, we were able to determine that, upon infection, U937 intracellular pH sensibly decreased. Hence, as a working hypothesis, we propose that *emrKY* regulation is finely tuned to respond to physico-chemical environment met by the bacterium when invading macrophages. Whether the repression observed in epithelial cells results from an active, cell-specific transcriptional repressing mechanism, i.e. actual induction of a negative regulator that respond to conditions met in epithelial cell, will pave the way for our next molecular investigations.

Extent of *emrKY* operon induction observed in the macrophage environment is very high (32-fold) and is in agreement with previous transcriptomic analysis^46^, suggesting a potential involvement of this EP in *Shigella* virulence. The results obtained in infection competition assays clearly indicated that EmrKY helps *Shigella* to better survive inside macrophages and, possibly, to achieve a successful host infection. Why and how can EmrKY contribute to *Shigella* survival in the macrophage environment? It has been reported that, in *E*. *coli*, mutations affecting *emrK* or *emrY* genes cause a hypersensitive phenotype to the lethal effect of several drugs, including mitomycin C, suggesting that EmrKY might be involved in safeguarding cells from DNA damaging agents^44^. Does the importance of EmrKY for multiplication in macrophages associate with its capacity to eliminate DNA damaging compound produced by the infected cells? This is one of many attractive possibilities our next study will focus on.

In conclusion, our molecular and cellular investigation allowed us to identify EmrKY as a new important actor within *Shigella* pathogenicity program. The present study is the first dealing with and demonstrating the involvement of EPs in *Shigella* invasive process and, undoubtedly, open new perspectives in the analysis of the complex interactions of *Shigella* with its host.

## Methods

### Construction of strains and plasmids

Bacterial strains and plasmids used in this study are listed in Table S1. M90T is a *S*. *flexneri* serotype 5 strain (GenBank CM001474.1). *E*. *coli* DH10b has been used as recipient in cloning experiments^47^. Strains M90T, M90T ∆*acrAB*, M90T ∆*evgA* and M90T ∆*evgS* were obtained using the one-step method of gene inactivation^48^. pZ*emrK-gfp* and *pZmdtJ-gfp* plasmids were obtained by cloning the *emrKY* or *mdtJI* regulatory regions upstream the *gfp* gene into pZEP08 vector^49^. p*ACacrAB* has been obtained by cloning the *acrAB* operon under the control of the ptac promoter carried by pGIP7 vector^50^. Mutations and constructs obtained in this study were verified by DNA sequencing (BioFab, Rome). Sequences of the oligonucleotides, designed on the basis of M90T genome, are reported in Table S2. PCR reactions were routinely performed using DreamTaq DNA polymerase (Fermentas) or the Pfu Taq DNA polymerase (Fermentas).

### Media, chemicals and growth conditions

Unless otherwise indicated, bacteria were grown aerobically in LB medium at 37 °C. When required cells were grown in M9 minimal medium supplemented with 10 μg/ml thiamine, 0.2% glucose, 0.5% casamino acids, 10 μg/ml nicotinic acid, 1 mM MgSO_4_ and 0.2 mM CaCl_2_ (M9 complete medium). Solid media contained 1.6% agar. Congo red was added (0.01% final concentration) to Trypticase soy agar to monitor the expression of the virulence phenotype prior infection assays. Antibiotics were used at the following concentrations: ampicillin 50 μg/ml; cloramphenicol 25 μg/ml; kanamycin 30 μg/ml; streptomycin 10 μg/ml. To assay *emrKY* and *mdtJI* promoter activity in response to different compounds, M90T strains carrying the *gfp* fusions (M90T pZ*emrK-gfp*, M90T pZ*mdtJ-gfp*) were grown in LB or M9 complete media at 37 °C as indicated. The overnight cultures were then washed and diluted 1:100 in the same media. The different compounds were added to cultures grown up to OD_600_ 0.2 in a final volume of 150 µl in each well of a 96-well plate. To induce oxidative stress paraquat (Sigma-Aldrich) or hydrogen peroxide (HPO) (Sigma-Aldrich), at final concentrations of 50 µM, 100 µM and 150 µM, were added to cultures grown in LB. Nitrosative stress was induced by supplementing LB cultures with NOC-5 (3-(Aminopropyl)-1-hydroxy-3-isopropyl-2-oxo-1-triazene) or NOC-7 (3-(2-Hydroxy-1-methyl-2-nitrosohydrazino)-N-methyl-1-propanamine) at final concentrations of 10 µM and 20 µM. To test the effect of alkali metals and osmolality, NaCl (0.1 M), KCl (0.1 or 0.2 M), sucrose (0.1 M or 0.2 M) or MgCl_2_ (0.05 or 0.1 M) were added to bacterial cultures grown in M9 complete medium.

To test the effect of pH variation, HCl or NaOH was added to the M9 complete medium to obtain the desired pH value. The fluorescence intensity of M90T strains harboring the *gfp* fusions was measured on Infinite 200 (TECAN). Fluorescence and OD_600_ were detected every 15 minutes throughout 3 hours. Fluorescence values were divided by the absorbance at 600 nm in order to normalize to bacterial cell concentration.

### Cell cultures and infections

Infection experiments were performed by using both U937 and Caco-2 cell lines. Human U937 cells (American Type Culture Collection, Manassas,VA) were grown in RF10 (RPMI 1640 [GIBCO] medium containing 10% heat-inactivated FBS [fetal bovine serum, Euroclone], 2 mM L-glutamine and PS [0.05 I.U./ml penicillin and 0.05 I.U./ml streptomycin]) at 37 °C in a humidified 5% CO_2_ atmosphere. For bacterial infection, cells were seeded in 6-well tissue culture plates (Falcon), at a density of 1.5 × 10^6^ cells/well, in RF10 supplemented with phorbol myristate acetate (PMA, 80 nM; Sigma) to induce macrophage differentiation. After two days, PMA containing medium was removed and cells left for further 4 days in RF10. Two hours before bacterial infection, RF10 was replaced with fresh RPMI containing only L-glutamine. Human Caco-2 epithelial cells (American Type Culture Collection, Manassas,VA) were grown in Dulbecco modified essential medium (DMEM) (GIBCO) containing 10% heat-inactivated FBS, 2 mM L-glutamine and PS, referred to as DF10, at 37 °C in a humidified 5% CO_2_ atmosphere. For bacterial infection, cells were seeded in 6-well tissue culture plates (Falcon), at a density of 4 × 10^5^ cells/well, in DF10. After 48 hours cells were serum-starved over-night in DMEM supplemented with 0.5% FBS, PS and L-glutamine (DF 0.5). Two hours before bacterial infection, DF 0.5 was replaced with fresh DMEM containing only L-glutamine. Both cell lines were infected at a MOI of 100. After addition of bacteria, plates were centrifuged for 15 min at 750 × g and incubated 30 min (U937) or 45 min (Caco-2) at 37 °C under 5% CO_2_ atmosphere to allow bacterial entry. Thereafter extracellular bacteria were removed by extensive washing with phosphate-buffered saline (PBS). This point was taken as time zero (T0). Fresh medium (RPMI 1640 or DMEM) containing gentamicin (100 μg/ml) was added to each plate to kill extracellular bacteria, and infected cells were incubated at 37 °C for up to 3 h. To visualize GFP expression in M90T and M90T ∆*evgA* harboring *emrK-gfp* fusion during the infection process, samples were fixed with 4% paraformaldehyde for 30 min and the DNA of bacteria and macrophages was stained with DAPI. Cells were examined using a Leica DMRE fluorescence microscope equipped with 20×, 40× and 100 × lenses. Single images were recorded on a Leica DF 420 camera and processed using the Qwin software (Leica Microsystem).

### RNA isolation and Real Time PCR

To monitor the expression of *emrK* and/or *evgA* during standard bacterial growth, M90T, M90T *ΔevgA* or M90T *ΔevgS* were grown in M9 complete medium until OD_600_ 0.2. Cultures were then supplemented with KCl and/or HCl to obtain pH 6 and RNA was extracted from strains grown until OD_600_ 0.5 as previously described^29^. To monitor gene expression during host cell infection, one 6-well tissue culture plate was considered for each time point to maximize the yield of intracellular bacteria. RNA was extracted from intracellular bacteria recovered after lysing infected cells with 1% Triton X-100 (Sigma) for 5 min. The lysate was diluted 1:2 with PBS to decrease Triton concentration before proceeding with RNA extraction. Two μg of total RNA were treated with DNAse I and then retro-transcribed using the High Capacity cDNA Reverse Transcription Kit (Applied Biosystems) following the manufacturer’s instruction. qRT-PCR was performed in a 30 μl reaction mix containing 2 μl cDNA using Power SYBR Green PCR Master Mix (Applied Biosystems) on a 7300 Real-Time PCR System (Applied Biosystems). At least three wells were run for each sample. Relative quantification was performed using the comparative cycle threshold (2^−ΔΔCt^) method^51^. Primers for the *nusA* transcript (endogenous control) and target transcripts were designed with the aid of the Primer Express software v2.0 and experimentally validated for suitability for the 2^−ΔΔCt^ method. All primers used are listed in Table S2. In the case of EP systems consisting of more than one protein (AcrAB, EmrAB, EmrKY, MacAB, MdtJI) encoded by genes clustered in a single operon, we monitored the transcription of the promoter-proximal gene.

### Measurement of cytosolic pH

U937 cells were seeded in 96-well tissue culture plates at a density of 5 × 10^4^ cells/well, differentiated and infected as described above. At 3 hours p.i. mock and *S*. *flexneri* M90T infected cells were loaded with pHrodo® Green AM intracellular pH indicator (ThermoFisher Scientific) according to the manufacturer’s instructions. After washing, fluorescence intensity was monitored by using the Victor multilabel counter (PerkinElmer). Afterwards, standard curves were created by incubating each sample with four different calibrated pH solutions (Intracellular pH Calibration buffer kit, ThermoFisher Scientific) and used to pinpoint the intracellular pH associated with the experimental conditions. Samples and calibrations were run in triplicate.

### Competition assay

The relative survival of *S*. *flexneri* M90T and M90T *ΔemrKY* was tested by co-infecting U937 cells with both strains. Bacteria were grown separately at 37 °C in LB to OD_600_ 0.6, then the two cultures were mixed, centrifuged and resuspended in RPMI 1640 containing 2mM L-glutamine. Macrophage infection was carried out as described above. Bacterial survival was monitored at time zero, and at 1, 2 and 3 hours p.i. At each time point, host cells were lysed with 1% Triton X-100, intracellular bacteria were collected, washed and resuspended in PBS. Serial dilutions of the bacterial suspension were plated on LB agar and, one day late, replicated on LB agar plates with or without 30 ug/ml kanamycin. To calculate the Competitive index (C.I.), the ratio of strains M90T *ΔemrKY*/M90T recovered at each time point from the infected cultures was determined and then normalized by dividing by the corresponding ratio in the initial inoculum.

### Statistical analyses

The statistical differences between treatments were determined using Microsoft Excel by calculating the p values derived from a one-tailed ANOVA or, when indicated, a two-tailed t-test.

## Supplementary information

Figure S1, Table S1 and Table S2
