# Author Correction: The MFS efflux pump EmrKY contributes to the survival of *Shigella* within macrophages

**DOI:** 10.1038/s41598-019-44357-2

**Published:** 2019-05-22

**Authors:** Martina Pasqua, Milena Grossi, Sara Scinicariello, Laurent Aussel, Frédéric Barras, Bianca Colonna, Gianni Prosseda

**Affiliations:** 1 Istituto Pasteur Italia, Dipartimento di Biologia e Biotecnologie “C. Darwin”, Sapienza Università di Roma, Rome, Italy; 2 Aix-Marseille Univ, CNRS, Laboratoire de Chimie Bactérienne, Institut de Microbiologie de la Méditerranée, Marseille, France; 3 Institut Pasteur, Department of Microbiology, Paris, France

Correction to: *Scientific Reports* 10.1038/s41598-019-39749-3, published online 27 February 2019

In this Article, Figure 1a is a duplication of Figure 1b. The correct Figure 1 appears below as Figure 1.

**Figure 1:**
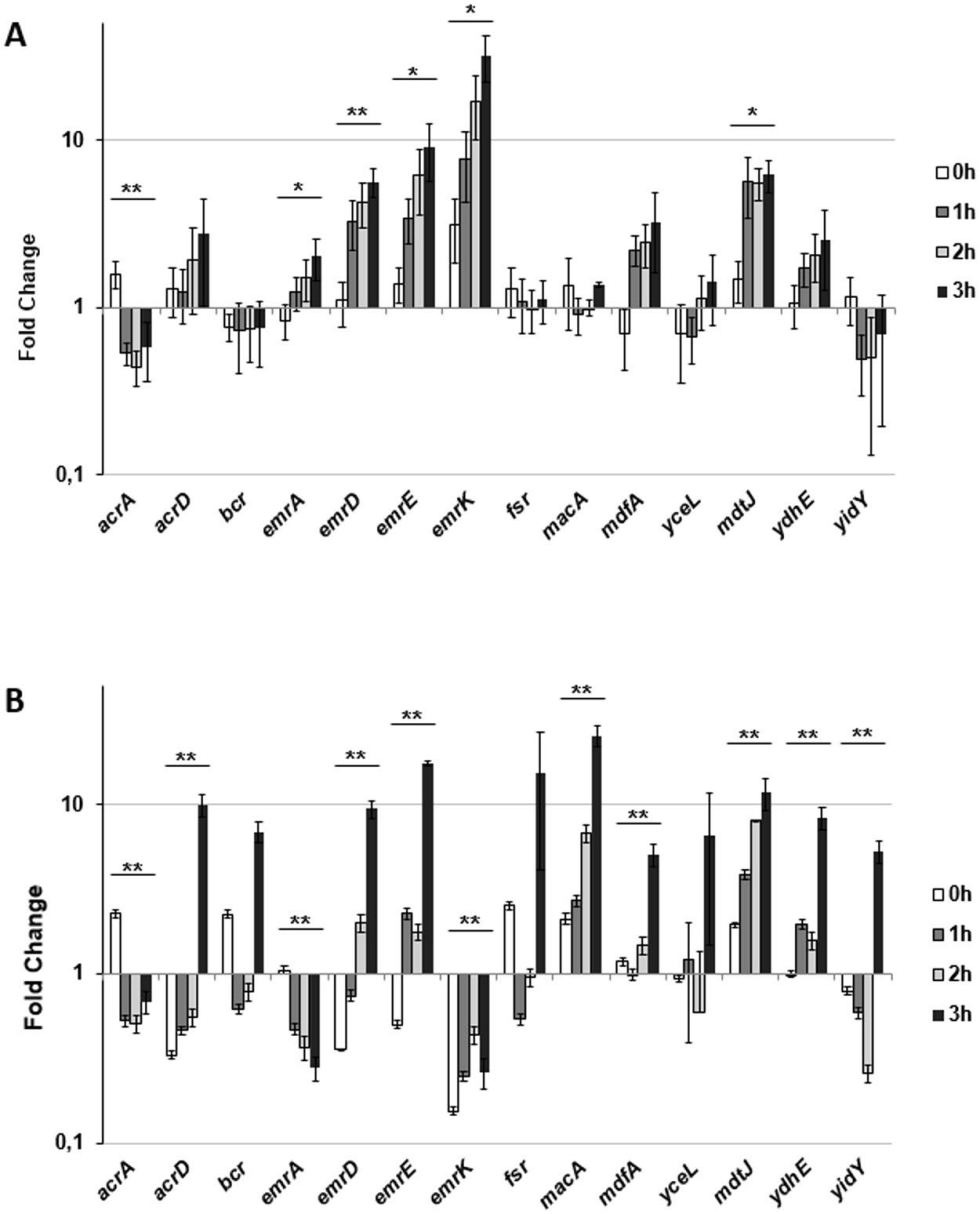
Relative efflux pumps encoding gene transcription during *S. flexneri* infection of (**A**) U937 and (**B**) Caco-2. Quantitative analysis of 14 efflux pump transcripts was performed by means of Q-Real Time PCR assay. Total RNA was extracted from intracellular *S. flexneri* M90T bacteria at various time points p.i., from 0 h (corresponding to bacterial adhesion to the target cells, see MM) up to 3 h. Each infection was repeated three times and at least three wells were run for each sample. The x axis indicates the expression fold-change (RQ value) for each gene on a logarithmic scale. The results are shown relative to the expression of each gene in bacteria grown in RPMI (**A**) or DMEM (**B**) set to 1.00. Statistical significance was determined by a one-tailed ANOVA, and p values are as follows: *p < 0.05, **p < 0.01. Error bars represent SD.

