# Supplementary material for: The MFS efflux pump EmrKY contributes to the survival of Shigella within macrophages
Source: Sci Rep. 2019 Feb 27;9:2906. doi: 10.1038/s41598-019-39749-3 (PMC6393483; doi:10.1038/s41598-019-39749-3)
Supplement: Supplementary file 1 — Figure S1, Table S1 and Table S2 [file 41598_2019_39749_MOESM1_ESM.pdf]

The MFS efflux pump EmrKY contributes to the survival of *Shigella* within macrophages

Pasqua M., Grossi M., Scinicariello S., Aussel L., Barras F., Colonna B. and Prosseda G.

Fig S1

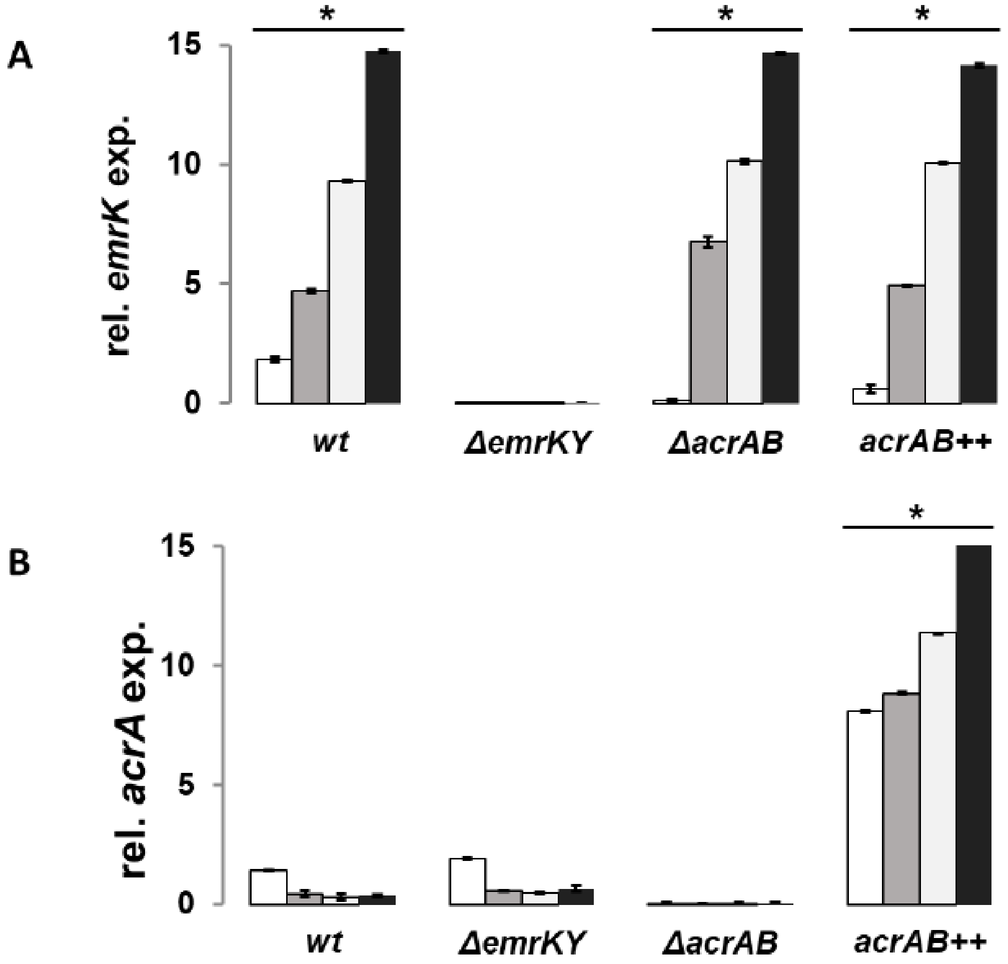

Figure S1. *emrK* expression during *S. flexneri* infection of U937 is independent from *acrA* transcription and *vice versa*. Quantitative analysis of (A) *emrK* and (B) *acrA* transcripts was performed by Real Time PCR assay using RNA extracted from intracellular S.*flexneri* M90T and its derivatives lacking EmrKY (M90T *ΔemrKY*) or AcrAB (M90T *ΔacrAB*) EP or carrying the pAC*acrAB* plasmid. The *emrK* and *acrA* expression was followed in each infecting strain from 0h up to 3h post-infection. At least three experiments were carried out and the results are shown relative to the expression in bacteria grown in RPMI. Statistical significance was determined by a one-tailed ANOVA, and p values are as follows: * $p<0.05$, ** $p<0.01$. Error bars represent SD.

The MFS efflux pump EmrKY contributes to the survival of *Shigella* within macrophages

Pasqua M., Grossi M., Scinicariello S., Aussel L., Barras F., Colonna B. and Prosseda G.

Table S1. Strains and plasmids used in this study

**Strain**	**Relevant characteristics**	**Source/Reference**
DH10b	*E. coli K12*	[44]
M90T	*S. flexneri 5a*	[30]
M90T Δ*mdtJI*	M90T mutant defective in *mdtJI* operon	[27]
M90T Δ*emrKY*	M90T mutant defective in *emrKY* operon	This study
M90T Δ*acrAB*	M90T mutant defective in *acrAB* operon	This study
M90T Δ*evgA*	M90T mutant defective in *evgA* gene	This study
M90T Δ*evgS*	M90T mutant defective in *evgS* gene	This study
M90T Δ*fnr*	M90T mutant defective in *fnr* gene	This study
**Plasmid name**	**Relevant characteristics**	**Source/Reference**
pKD4	*kan*-containing plasmid, template for PCR	[45]
pKD46	Red recombinase expression plasmid	[45]
pGIP7	pACYC184 derivative carrying Ptac promoter	[47]
pZEP08	GFP reporter plasmid; Ap^R, Cm^R	[46]
pAC*acrAB*	pGIP7 derivative carrying *S. flexneri acrAB* operon	This study
pZ*emrKYgfp*	pZEP08 derivative carrying the *S. flexneri emrKY* promoter region	This study
pZ*mdtJIgfp*	pZEP08 derivative carrying the *S. flexneri mdtJI* promoter region	This study

The MFS efflux pump EmrKY contributes to the survival of *Shigella* within macrophages

Pasqua M., Grossi M., Scinicariello S., Aussel L., Barras F., Colonna B. and Prosseda G.

Table S2. Oligonucleotides used in this study

Name	Sequence (5'-3')	Used for
KYF	TCACTGGATAAAACTGATGCCACTATCGCACTC AATAGTGTAGGCTGGAGCTGCTTC	*emrKY* deletion
KYR	TGTCACAGAACGCCAGTAATAGCAAACCGCAT ACATCAATTCCGGGGATCCGTCGACC	*emrKY* mutation
ABF	ACTTTTGACCATTGACCAATTTGAAATCGGACA CTCGAGGTTTACATATGTGTAGGCTGGAGCTGC TTCG	*acrAB* mutation
ABR	TAGGCATGTCTTAACGGCTCCTGTTTAAGTTAA GACTTGGACTGTTCAGGATTCCGGGGATCCGTC GACC	*acrAB* mutation
AF	AAAGACCACAACAGAGAAGAAAAAAATAGGG TAAAAACTTCATATGAATATCCTCCTTA	*evgA* mutation
AR	CTGTATTACTACAGGGAGAAGGGAGATGCTTC ATTGCAAAGTGTAGGCTGGAGCTGCTTC	*evgA* mutation
SF	ATCTTTACACATTCGCGCAACGTAACAAAATC GGCTAATCGTGTAGGCTGGAGCTGCTTC	*evgS* mutation
SR	AAGATGTGCTGGTAAATAGCTCCCACATTAGA ACATTGTGGAATATCCTCCTTAGTTCC	*evgS* mutation
p*acrAB*F	NNN GGATCCATGACCAATTTGAAATC	Cloning *acrAB* genes
p*acrAB*R	NNN GGATCCATACGTGGTTAATACT	
KPF	NNNCCCGGGTTACGAATTGCTGCGATAGC	Cloning *emrKY* promoter
KPR	NNNTCTAGATGACCAATAGGCATAGGCAC	
JPF	NNNCCCGGGCTCGGCGGCAAAACGGATA	Cloning *mdtJI* promoter
JPR	NNNTCTAGAGACGCTCGCCCATTTCATTG	
acrAF	GTCTATCACCCTACGCGCTATCTT	Real Time PCR
acrAR	GCGCGCACGAACATACC	Real Time PCR
acrDF	GTACCCTGGCGATTTTTTCATT	Real Time PCR
acrDR	CGGTCACTCGCACATTCA	Real Time PCR
bcrF	TGTTTTTCTGTTCGTGATGAGGAT	Real Time PCR
bcrR	GGAACATATTTAACGCCGCCAAT	Real Time PCR
emrAF	CGTTCCAGCCACCAATATGTG	Real Time PCR
emrAR	CCGTAAATATCGGTGGTGATGG	Real Time PCR
emrDF	GTGGATCCCCGACTGGTTT	Real Time PCR
emrDR	CCCGGCACCGAAAAAGA	Real Time PCR

The MFS efflux pump EmrKY contributes to the survival of *Shigella* within macrophages

Pasqua M., Grossi M., Scinicariello S., Aussel L., Barras F., Colonna B. and Prosseda G.

emrEF	CACCAGGATGAAAACGGTGAGT	Real Time PCR
emrER	TGACCTCTGCAAGTATTGCGC	Real Time PCR
emrKF	GCGTTAAACGTACGGATATTAAGA	Real Time PCR
emrKR	ACTGTTTCGCCGACCTGAAC	Real Time PCR
fsrF	ACGCTGATTTTACCCTACGCC	Real Time PCR
fsrR	AGCGTAGACCAGAATGGCAGAG	Real Time PCR
macAF	CGACGCCGGAAAAGGTAAA	Real Time PCR
macAR	ATGCACTTGCGCGGTCATA	Real Time PCR
mdfAF	CTTGCTGTTAGCGCGTCTGA	Real Time PCR
yceLF	TTTTCACCCTGATTTGTCTGTTTTAT	Real Time PCR
yceLR	CAGCGAAAGCACTTAAGGTTTCA	Real Time PCR
mdtJF	TGATGAAAATTGCCGGGTTAA	Real Time PCR
mdtJR	CGCTTTACGGGTACCTGATTTTA	Real Time PCR
ydhEF	CCGGTTATCGCGCAATTAAAT	Real Time PCR
ydhER	GAAACCTTGTCGCACCTGATG	Real Time PCR
yidYF	TATCCCGCCGGGATTGATAT	Real Time PCR
yidYR	CGCTTCGCTGGCATTGA	Real Time PCR
nusAF	TGAAGCCGCACGTTATGAAG	Real Time PCR
nusAR	TCAACGTAATCGCCCAAGTT	Real Time PCR
evgAF	TCTTGCTATCGCAGCAATTCG	Real Time PCR
evgAR	AAGTGTTTCCACCCGCTGAAC	Real Time PCR
